# *Helicobacter pylori* seropositivity associates with hyperglycemia, but not obesity, in Danish children and adolescents

**DOI:** 10.1186/s12916-024-03591-w

**Published:** 2024-09-11

**Authors:** Sigri Kløve, Sara E. Stinson, Fie O. Romme, Julia Butt, Katrine B. Graversen, Morten A. V. Lund, Cilius E. Fonvig, Tim Waterboer, Guillermo I. Perez-Perez, Torben Hansen, Jens-Christian Holm, Sandra B. Andersen

**Affiliations:** 1 Center for Evolutionary Hologenomics, Globe Institute, Faculty of Health and Medical Sciences, University of Copenhagen, Copenhagen, 1353 Denmark; 2 Novo Nordisk Foundation Center for Basic Metabolic Research, Faculty of Health and Medical Sciences, University of Copenhagen, Copenhagen, 2200 Denmark; 3 Infections and Cancer Epidemiology, German Cancer Research Center, Heidelberg, Germany; 4 Department of Biomedical Sciences, Faculty of Health and Medical Sciences, University of Copenhagen, Copenhagen, 2200 Denmark; 5 The Children’s Obesity Clinic, accredited European Centre for Obesity Management, Department of Pediatrics, Holbæk Hospital, Holbæk, 4300 Denmark; 6 Department of Clinical Medicine, Faculty of Health and Medical Sciences, University of Copenhagen, Copenhagen, 2200 Denmark; 7 School of Medicine, New York University, New York, NY 10016 USA

**Keywords:** *Helicobacter pylori*, Obesity, Body mass index, Hyperglycemia, Cardiometabolic risk factors, Pediatric, Children, Adolescents

## Abstract

**Background:**

*Helicobacter pylori* colonizes the human stomach and may affect the inflammatory response, hormone production related to energy regulation, and gastrointestinal microbiota composition. Previous studies have explored a potential association between *H. pylori* infection and pediatric obesity with varying results. Considering the immunomodulatory effects of early-life infection with *H. pylori* that can confer beneficial effects, we hypothesized that we would find an inverse relationship between *H. pylori* seropositivity and obesity among Danish children and adolescents.

**Methods:**

We assessed *H. pylori* seroprevalence in 713 subjects from an obesity clinic cohort and 990 subjects from a population-based cohort, aged 6 to 19 years, and examined its association with obesity and other cardiometabolic risk factors.

**Results:**

No association was found between *H. pylori* and body mass index standard deviation score (BMI SDS). *H. pylori* seropositivity was, however, significantly associated with higher fasting plasma glucose levels and the prevalence of hyperglycemia.

**Conclusion:**

While we did not find an association between *H. pylori* seropositivity and BMI SDS, we observed a significant association with higher fasting plasma glucose levels and increased prevalence of hyperglycemia, suggesting that *H. pylori* infection may contribute to impaired glucose regulation in Danish children and adolescents.

**Graphical abstract:**

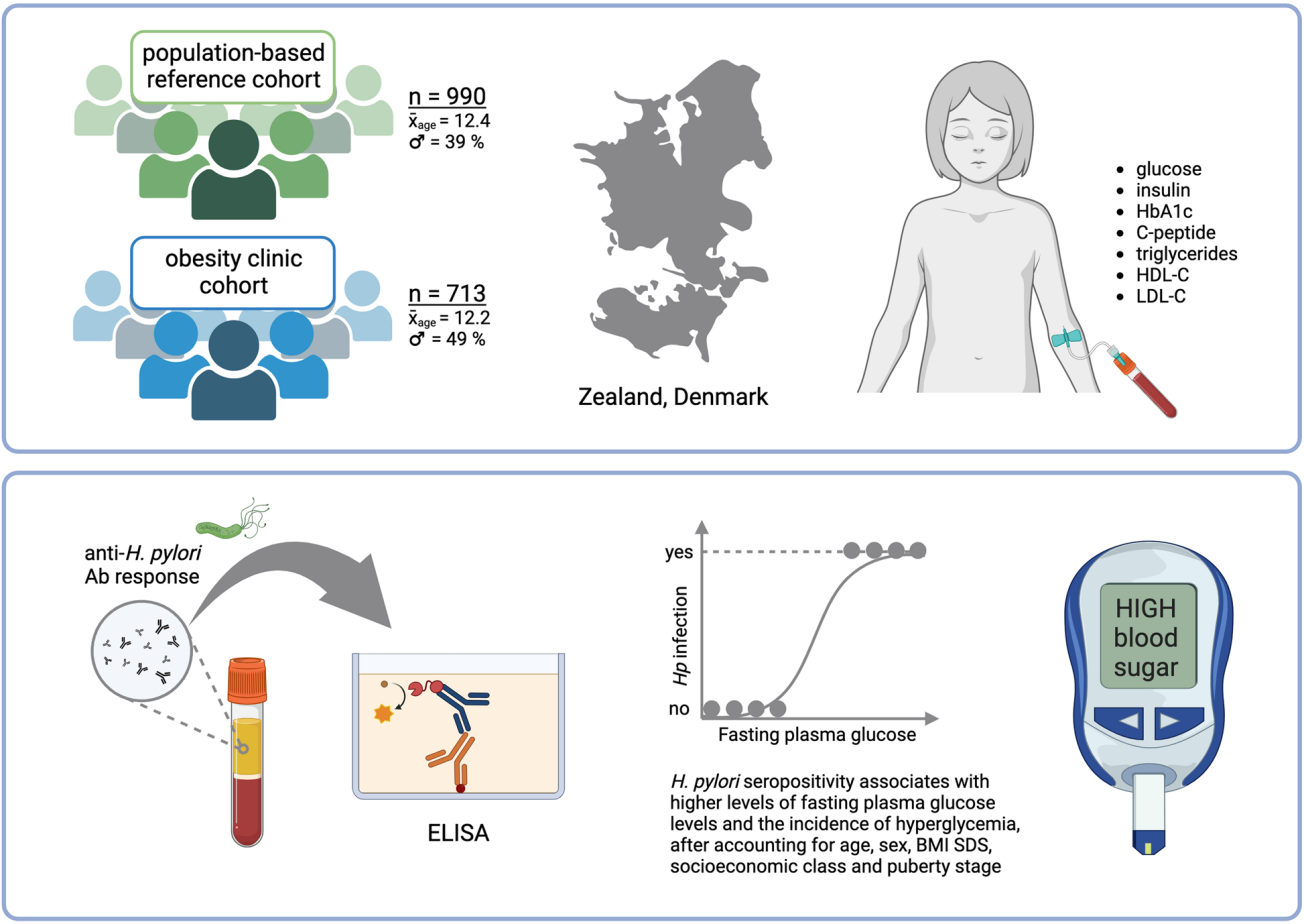

**Supplementary Information:**

The online version contains supplementary material available at 10.1186/s12916-024-03591-w.

## Background

Obesity is a complex disease defined by excessive or abnormal fat accumulation that may impair health [1]. Furthermore, obesity is characterized by chronic low-grade inflammation, which increases the risk of developing cardiometabolic complications [2]. The prevalence of obesity has reached epidemic proportions globally and continues to rise. Recently, the World Health Organization (WHO) reported that among children and adolescents, the global prevalence has increased from 8% in 1990 to 20% in 2022 [1]. For Denmark, the latest report on overweight and obesity has assessed the prevalence among children starting primary school (6–7 years old) to be 13% and that of adolescents (14–15 years old) to be higher at 18% [3].

The development of obesity is affected by a complex interplay between genetic, socioeconomic, and environmental factors. Emerging research suggests that the composition of the gastrointestinal microbiome also plays a role in obesity [4, 5]. Disruption of the early life microbiome, such as a caesarean section or antibiotic treatment, increases the risk of obesity later in life [6–8]. This could be the result of a less diverse microbiome potentially hindering the proper development of the host immune system and triggering the development of autoimmune diseases and obesity [9].

*Helicobacter pylori* (*H. pylori*) is an ancestral member of the gastric microbiome that has been associated with humans for at least 100,000 years [10]. While prevalence is declining in association with improving standards of living, *H. pylori* is estimated to colonize about half of the world’s population and is usually acquired during early childhood [11]. For most carriers, colonization remains asymptomatic but persists throughout the host’s lifetime if not treated. It is estimated that approximately 10% of individuals who are infected with *H. pylori* will develop peptic ulcer disease. Furthermore, depending on a combination of genetic and environmental factors, between 1 and 5% of infected individuals are considered to be at risk for developing gastric cancer [12]. It is currently not well understood what determines the outcome of a *H. pylori* infection, but age at acquisition [13], presence of virulence factors [14], and host-microbe genomic match [15] seem to play important roles. Mechanistic studies in mice have revealed that *H. pylori* has immunomodulatory effects that can confer protection against asthma and allergies, most robustly when the infection is established early in life [16, 17]. Similar results were found in human correlative studies [18, 19].

*H. pylori* has been investigated in relation to pediatric obesity, with two studies showing lower *H. pylori* seropositivity in children and adolescents with obesity [20, 21]. Other studies found no association [22, 23]. One European study demonstrated an increase in body mass index (BMI) after eradication of *H. pylori* [24], similar to an earlier finding in adults [25]. Results from studies on adults show conflicting results concerning an effect of *H. pylori* on obesity [26]. This may be attributed to confounding variables, as both obesity and *H. pylori* infection status is known to correlate with ethnicity, socioeconomic status, age, and sex in both older and recent studies [27–32].

In the present study, we aimed to investigate the relationship between *H. pylori* seropositivity and obesity in Danish children and adolescents and its association with cardiometabolic risk factors. Based on *H. pylori*’s immunomodulatory properties, along with previous findings from pediatric cohorts [20, 21, 33], we hypothesized that *H. pylori* seropositivity would be inversely associated with obesity and cardiometabolic risk factors.

## Methods

### Study populations

We used a random subset of individuals from The HOLBAEK Study, which has been described elsewhere [34, 35]. Briefly, serum samples derived from two groups of children and adolescents: (a) an obesity clinic group (*n* = 713) consisting of individuals with a BMI standardized deviation score (SDS) > 90th percentile (BMI SDS > 1.28) according to Danish reference values [36], who followed a multifaceted childhood obesity management program at Holbæk Hospital, and (b) a population-based reference group (*n* = 990) consisting of subjects recruited from schools in the same geographical area. Participants from both groups were enrolled in the HOLBAEK study between 2010 and 2019. The study participants have been thoroughly characterized in relation to their age, sex, socioeconomic status, and various cardiometabolic risk factors. The characteristics of the full obesity clinic and population-based cohorts have been described elsewhere [35]. For the subset used here, the groups did not differ in age, but there were more boys in the obesity clinic cohort than in the population-based reference cohort (49% vs 39%; Additional file 1: Table S1). In the population-based reference group, there were 184 subjects with obesity (defined as BMI SDS > 90th percentile and 52 subjects with underweight (defined as BMI SDS < 10th percentile). In the present study, exclusion criteria were children under six (*n* = 10), originally included for a supplementary study [37], and over 19 years of age (*n* = 13) originally included to avoid loss into the adult care system. Additionally, subjects who fulfilled type 2 diabetes criteria [38] based on blood samples showing fasting plasma glucose > 7.0 mmol/L (*n* = 2) and/or glycated hemoglobin (HbA1c) > 48 mmol/mol (*n* = 2) were excluded. The subset of individuals from which samples were available for screening were predefined; however, sample size calculations predicted that a seropositivity rate between 0.1 and 0.2 would allow us to detect a difference of 50% compared to 40% between groups in individuals defined as obese with a sample size of 1398 to 2347 individuals with alpha = 0.05 and beta = 0.2 (https://clincalc.com/stats/samplesize.aspx).

### Ethics

In accordance with the Declaration of Helsinki, participants provided informed consent. For individuals below 18 years of age, written consent was acquired from their parents or legal guardians, whereas those 18 years or older provided their own written consent. The study received approval from the Scientific Ethics Committee of Region Zealand, Denmark (protocol No. SJ-104), and the Danish Data Protection Agency. The HOLBAEK Study is registered at ClinicalTrials.gov (NCT00928473).

### Anthropometrics

Anthropometric data (height, weight, and waist measurements) was collected during routine clinical examinations in the obesity clinic group. Meanwhile, the population-based group underwent assessments in a mobile laboratory, administered by trained medical professionals. Waist-to-height ratio (WtHR) SDS was calculated based on age- and sex-specific reference values [39]. Whole-body dual-energy x-ray absorptiometry (DEXA) was performed to quantify body fat % in a subset from both the obesity clinic (*n* = 650) and population-based (*n* = 71) groups, using a GE Lunar iDXA (ME + 200,179, GE Healthcare) as earlier described [40]. Body fat % SDS was calculated based on age- and sex-specific reference values [41]. Puberty stage was assessed by the Tanner stage classification method [42, 43]. In the obesity clinic group, a pediatrician evaluated Tanner stages based on breast development in girls and gonad development in boys. In the population-based group, a self-assessment approach was utilized, where individuals used a standard questionnaire with picture patterns for recognition.

### Genotyping

Participant genotyping was carried out as described previously [44, 45]. Out of the 1703 included participants, 1379 had been genotyped. In brief, DNA was extracted and genotyped by using Illumina Infinium HumanCoreExome-12 v1.0 and HumanCoreExome-24 v1.1 Beadchips, with the Illumina HiScan system. Genotype calling was performed using the Genotyping module (version 1.9.4) of GenomeStudio software (version 2011.1; Illumina). Subsequently, the data were phased using EAGLE2 (version 2.0.5) and imputed to the Haplotype Reference Consortium (HRC, r1.1) using PBWT on the Sanger server. Individuals not of European descent were identified through Principal Component Analysis using ancestry informative markers. Study samples were classified as non-European when their Euclidean distance from the center exceeded a radius greater than 1.5 times the maximum Euclidean distance of the European reference samples from the 1000 Genomes dataset [46].

### Biochemical analyses

Serum was sampled as previously described [47]. Briefly, venous blood samples were collected from 7 to 9 am in ice-cold ethylenediaminetetraacetic acid (EDTA) tubes following an overnight fast of minimum 8 h and separated by centrifugation within 20 min. Samples were stored at − 80 °C until further analysis. Serum antibody responses to *H. pylori* were determined by whole-cell enzyme-linked immunosorbent assay (ELISA) as previously described [48] with a few modifications. *H. pylori* whole-cell antigen was collected from the *H. pylori* strain PMSS1 [49] grown on blood agar plates for 48 h under microaerophilic conditions and lysed with the B-PER™ Bacterial Protein Extraction Kit (Fisher Scientific). Antigens were coated to microtiter wells (Immulon 2 HB) in carbonate-bicarbonate buffer (0.05M, pH 9.6) to a final concentration of 0.1 mg/well at room temperature (RT) overnight. The following morning, plates were blocked for 3 h at 37 °C with 200 µL phosphate-buffered saline (PBS, pH 7.2–7.4) containing 0.5% Tween 20 and 0.1% gelatin (PBSTG). Subsequently, plates were washed twice with 250 µL PBS containing 0.5% Tween 20 (PBST). Serum samples were diluted 1:200 in 100 µL PBSTG containing 0.5% bovine γ-globulin (PBSTGG) and analyzed in duplicate. On each plate, four positive serum controls and four negative serum controls were included, along with two blanks and four calibrator samples from the *H. pylori* IgG Test System (Zeus Scientific). The positive serum control consisted of a pool of 30 samples from the HOLBAEK study that were determined as seropositive with the *H. pylori* IgG Test System (Zeus Scientific). After 1 h incubation at 37 °C, plates were washed three times with 250 µL PBST per well. Next, peroxidase labeled antibody (polyclonal IgG Fc Cross-Adsorbed Goat anti-Human, Invitrogen) was diluted 1:500 in 100 µL PBST containing 0.1% bovine γ-globulin and 1% bovine serum albumin (PBSTGB) and incubated for 1 h at 37 °C. Finally, plates were washed five times with 250 µL PBST and then incubated with 100 µL substrate solution (Mcllvains buffer pH 4.6 with 0.1% ABTS and 0.16% H_2_O_2_ [30%]) for 5 min at RT in the dark before absorbance was measured at 420 nm. Optical density (OD) ratio was calculated by standardizing the absorbance values with the mean calibrator value.

### Determination of seropositivity cut-off value

Based on the commercial adult-based calibrator sample, samples with an OD ratio ≥ 1 should be classified as positive (Zeus Scientific). However, as children have physiologically lower antibody titers than adults, previous studies have demonstrated lower sensitivity and specificity of ELISAs in pediatric populations where adult-derived cut-off values were employed [50, 51]. Consequently, we aimed to validate the adult-derived cut-off value for the present study population through an independent assay. To achieve this, 60 samples were subjected to analysis by multiplex serology. We categorized samples with OD ratios between 0.2 and 1 as intermediate, determining the cut-off value of 0.2 by adding three standard deviations to the mean OD of the four negative controls included on each plate [52], and then averaging across all plates. We selected 44 samples with intermediate OD ratios, evenly distributed between 0.2 and 1. As controls, 6 negative samples (OD ratio < 0.2) and 10 positive samples (OD ratio ≥ 1) were included. It was ensured that cohort, age, and sex distribution of the subset resembled the total study population. *H. pylori* multiplex serology was performed as previously described [53]. Briefly, 12 *H. pylori* proteins were recombinantly expressed as glutathione-S-transferase (GST)-tagged proteins and affinity-purified on glutathione-casein coated beads with distinct internal fluorescence (Luminex Corp., Austin, TX, USA). Sera were incubated with a suspension array of the antigen-loaded beads in a 1:100 serum dilution and bound serum IgG serum antibodies were labeled using a biotinylated goat anti-human IgG secondary antibody (Jackson ImmunoResearch, Ely, UK) and Streptavidin-R-phycoerythrin (Moss Inc., Pasadena, MD, USA). A Luminex analyzer (Luminex Corp., Austin, TX, USA) then identifies the bead type and consequently the bound antigen as well as quantifies the amount of bound serum antibodies given as the median fluorescence intensity of 100 beads per type measured. Pre-defined antigen-specific cut-offs for *H. pylori* antigens were applied (Additional file 1: Table S2) and quality assured by the visual inflection point method, and samples were classified as overall *H. pylori* seropositive when being positive to any four or more of the included *H. pylori* antigens as described previously [54]. All eight samples identified as seropositive by multiplex serology were also classified as positive or intermediate in the ELISA assay (Additional file 1: Table S3). However, the seropositivity rate was overall lower in multiplex serology, compared to ELISA, and we consequently decided to base the analyses in this study on the adult-based ELISA cut-off value (OD ratio ≥ 1).

### Definition of variables

For the purpose of this study, obesity was defined as a BMI SDS > 90th percentile. Definition of the remaining cardiometabolic risk factors (hyperglycemia, hypertension, dyslipidemia and insulin resistance) has been described elsewhere [35]. Age was included as a continuous variable. Tanner stage was dichotomized, where Tanner stage 1 was classified as pre-pubertal and Tanner 2–5 was classified as pubertal/post-pubertal. The socioeconomic status variable was categorized into five levels according to the working status of the parents or legal guardians, based on the Statistics Denmark’s national classification [55], with 1 representing the highest level (general directors and others), 2 higher middle level (managers, company owners and others), 3 middle level (functionaries, skilled workers and others), 4 lower middle level (workers, students and others), and 5 lowest level (unemployed). In this study, socioeconomic status level 4 and 5 were merged to increase statistical power because of small sample sizes. Ethnicity as self-reported country of origin and ancestry (available for 1558 out of 1703 participants) was categorized as Danish or non-Danish.

### Statistical analyses

All statistical analyses were performed in R studio (version 4.3.3). The obesity clinic group and the population-based reference group were merged to increase statistical power and to account for instances where individuals in the population-based reference group had a BMI SDS > 90th percentile. Continuous variables were characterized as means with standard deviation (SD) and categorical variables were described as frequencies and percentages (%). *P* values for continuous variables comparing two groups were calculated using Wilcoxon rank sum tests, while categorical variables were calculated using *χ*^2^ tests. Generalized logistic regression models were used to calculate odds ratios (OR) and 95% confidence intervals (CI) with *H. pylori* infection status (0/1) as predictor of cardiometabolic risk factors (obesity, hypertension, hyperglycemia, insulin resistance, and dyslipidemia) with the adjustment of age, sex, and BMI SDS (except when the outcome was obesity). A second model was calculated with the additional adjustment of socioeconomic status (excluding 172 individuals with missing values), and a third model was additionally adjusted for puberty stage (excluding 371 individuals with missing values) to account for transient insulin resistance [56]. Linear regression models were used to calculate the effect size (*β*) and 95% confidence intervals (CI) with *H. pylori* infection status (0/1) as predictor of BMI SDS, body fat % SDS, WtHR SDS, fasting plasma glucose, fasting serum insulin, fasting whole blood HbA1c, and fasting serum C-peptide (all variables were log10 transformed and *z*-scored prior to analysis, except BMI SDS, body fat % SDS, and WtHR SDS) with the adjustment of age, sex, and BMI SDS (except when the outcome was BMI SDS, body fat % SDS, or WtHR SDS). A second model was additionally adjusted for socioeconomic status and a third model for puberty stage. Missing values were removed prior to the analysis and the number of samples removed is specified in Tables 2 and 3. Whenever *H. pylori* demonstrated a significant effect on the outcome, we examined whether sex, BMI SDS, socioeconomic status, or puberty stage (if these variables exhibited significance as main effects) acted as an effect modifier by including an interaction term and evaluating its significance.

## Results

### *H. pylori* seroprevalence and characteristics of the participants stratified by *H. pylori* seropositivity status

Out of the 1703 included subjects, we found 253 individuals with *H. pylori* seropositivity by ELISA, resulting in a prevalence of 14.8%. The frequency of seropositivity increased with age (Additional file 1: Fig. S1), and individuals with *H. pylori* seropositivity were older than those with seronegativity (Table 1, *p* = 0.04). There was a higher proportion of those with seropositivity than seronegativity in the lowest socioeconomic status (*p* = 0.015). Subjects with *H. pylori* seropositivity constituted a larger proportion in the hyperglycemic group (*p* < 0.001) and had higher fasting plasma glucose (*p* = 0.006), serum insulin (*p* = 0.018) and homeostatic model assessment for insulin resistance (HOMA-IR) concentrations (*p* = 0.007) than subjects expressing seronegativity. When analyzing the two cohorts separately, we observed that the association between *H. pylori* seropositivity and socioeconomic status was only statistically significant within the obesity clinic cohort (Additional file 1: Table S4). Meanwhile, the association between *H. pylori* seropositivity and hyperglycemia, as well as the correlations with fasting plasma glucose and HOMA-IR, were only significant in the population-based reference cohort (Additional file 1: Table S5). Within the population-based reference cohort, we found a significant association between *H. pylori* seropositivity and passive smoking (Additional file 1: Table S5). Overall, 71 individuals were identified with genotypes other than European of which 26.7% exhibited *H. pylori* seropositivity and 81.7% had obesity. Excluding the 71 subjects with non-European ethnicities from the dataset resulted in a weaker and non-significant association between *H. pylori* seropositivity and age as well as socioeconomic status (Additional file 1: Table S6). Furthermore, after exclusion of an additional 159 subjects who self-reported their ethnicity as non-Danish, we found a significant association solely between *H. pylori* seropositivity and fasting plasma glucose levels (Additional file 1: Table S7).

**Table 1:** Descriptive characteristics of study population stratified by *Helicobacter pylori* infection status

	*H. pylori*^*1*^		
**Characteristic**^a^	**Seronegative (*n* = 1450)**	**Seropositive (*n* = 253)**	*p* value^2^
Age	12.2 (3.5)	12.8 (3.4)	0.044
Sex			0.2
Male	630 (43%)	98 (39%)	
Female	820 (57%)	155 (61%)	
Socioeconomic status			0.015
1	430 (33%)	71 (32%)	
2	326 (25%)	40 (18%)	
3	327 (25%)	56 (25%)	
4 and 5	225 (17%)	56 (25%)	
Passive smoking			0.2
No	960 (66%)	156 (62%)	
Yes	490 (34%)	97 (38%)	
Puberty stage^3^			0.3
Pre-pubertal	387 (34%)	60 (30%)	
Post-pubertal	745 (66%)	140 (70%)	
BMI SDS	1.43 (1.62)	1.47 (1.58)	0.8
Plasma HDL-C (mmol/L)	1.40 (0.35)	1.39 (0.37)	0.3
Plasma LDL-C (mmol/L)	2.17 (0.67)	2.21 (0.64)	0.3
Plasma triglycerides (mmol/L)	0.84 (0.49)	0.86 (0.45)	0.5
Plasma glucose (mmol/L)	4.97 (0.39)	5.05 (0.37)	0.006
Plasma glucagon (pmol/L)	8.0 (4.3)	7.9 (4.3)	0.5
Serum insulin (pmol/L)	79 (56)	83 (52)	0.018
HOMA-IR (mIU/L)	2.90 (2.00)	3.06 (1.69)	0.007
Serum C-peptide (nmol/L)	0.65 (0.30)	0.69 (0.33)	0.052
Whole blood HbA1c (mmol/mol)	33.13 (2.69)	33.46 (2.89)	0.2
Obesity			> 0.9
No	686 (47%)	121 (48%)	
Yes	764 (53%)	132 (52%)	
Hyperglycemia			0.001
No	1266 (93%)	200 (86%)	
Yes	98 (7.2%)	32 (14%)	
Insulin resistance			0.3
No	1092 (82%)	178 (79%)	
Yes	240 (18%)	48 (21%)	
Dyslipidemia			0.8
No	1126 (82%)	194 (83%)	
Yes	254 (18%)	41 (17%)	
Hypertension			0.8
No	1293 (90%)	223 (90%)	
Yes	139 (9.7%)	26 (10%)	

^1^Mean (SD); *n* (%)

^2^Wilcoxon rank sum test; Pearson’s *χ*^2^ test

^3^Puberty stage defined as pre-pubertal (Tanner stage 1) or pubertal (Tanner stages 2–5)

^a^*Abbreviations*: *BMI SDS *Body mass index standard deviation score, *HDL-C *High-density lipoprotein cholesterol, *LDL-C *Low-density lipoprotein cholesterol, *HOMA-IR *Homeostasis model assessment of insulin resistance, *HbA1c *Glycated hemoglobin

### *H. pylori* infection as a predictor for cardiometabolic risk factors

*H. pylori* seropositivity was associated with a higher incidence of hyperglycemia (Table 2, model 1, OR = 2.03, *p* = 0.001), when adjusted for age, sex, and BMI SDS and also after additional adjustment for socioeconomic status (model 2, OR = 1.96, *p* = 0.005) and puberty stage (model 3, OR = 2.36, *p* < 0.001). Among the cardiometabolic risk features tested, *H. pylori* seropositivity was associated with higher levels of fasting plasma glucose (Table 3, model 1, β = 0.2, *p* = 0.005) when adjusted for age, sex, and BMI SDS but also after additional adjustment of socioeconomic status (model 2, β = 0.16, *p* = 0.03) and puberty stage (model 3, β = 0.2, *p* = 0.016). We also examined the effect of including interaction terms between *H. pylori* seropositivity and sex, BMI SDS, socioeconomic status, and puberty stage respectively. However, none of these interaction terms had a significant effect on either the prevalence of hyperglycemia or fasting plasma glucose levels (Additional file 1: Table S8). We used a complete case approach excluding missing values, so to explore if this caused a bias, we tested if there was a significant difference in seropositivity or hyperglycemia between the included individuals and those excluded due to missing information on socioeconomic status. This was not the case (model 2, 172 individuals, seropositivity: *χ*^2^ = 0.55, df = 1, *p* = 0.46; hyperglycemia: *χ*^2^ = 1.53, df = 1, *p* = 0.22) or puberty stage (model 3, 371 individuals, seropositivity: *χ*^2^ = 0.05, df = 1, *p* = 0.83; hyperglycemia: *χ*^2^ = 0.18, df = 1, *p* = 0.67).

**Table 2:** Estimated odds ratios (ORs) with 95% confidence intervals (CI) for associations of *Helicobacter pylori* seropositivity as an indicator of categorical (yes/no) cardiometabolic risk factors

	**Model 1**^**a**^			**Model 2**^**b**^			**Model 3**^**c**^		
	n*	**OR (95% CI)**	***p***	n*	**OR (95% CI)**	***p***	n*	**OR (95% CI)**	***p***
Obesity	1703	1.01 (0.77; 1.32)	0.94	1531	0.85 (0.62; 1.17)	0.32	1209	0.94 (0.65; 1.35)	0.73
Hyperglycemia	1596	2.03 (1.30; 3.09)	0.001	1436	1.96 (1.20; 3.10)	0.005	1151	2.36 (1.40; 3.88)	0.001
Hypertension	1681	1.08 (0.67; 1.69)	0.73	1514	0.86 (0.50; 1.43)	0.58	1199	0.96 (0.54; 1.64)	0.90
Dyslipidemia	1615	0.87 (0.58; 1.29)	0.50	1455	0.88 (0.57; 1.35)	0.57	1164	0.79 (0.47; 1.27)	0.34
Insulin resistance	1558	1.26 (0.85; 1.84)	0.24	1404	1.13 (0.73; 1.72)	0.57	1122	1.36 (0.84; 2.17)	0.20

^a^Adjusted for age, sex, body mass index standardized deviation score(except for the outcome “obesity”)

^b^Additional adjustment for socioeconomic status

^c^Additional adjustment for puberty stage, defined as pre-pubertal (Tanner stage 1) or pubertal (Tanner stages 2–5)

^*^Sample size after removal of missing values

**Table 3:** Standardized coefficient (*β*) estimates with 95% confidence intervals (CI) for associations of *Helicobacter pylori* seropositivity as an indicator of continuous cardiometabolic risk factors. Outcome variables were log10-transformed and *z*-scored, except for body mass index (BMI) standardized deviation score (SDS), body fat % SDS, and waist-to-height ratio (WtHR) SDS

	**Model 1**^**a**^			**Model 2**^**b**^			**Model 3**^**c**^		
	n*	**β (95% CI)**	***p***	n*	**β (95% CI)**	***p***	n*	**β (95% CI)**	***p***
BMI SDS	1703	0.07 (− 0.14; 0.29)	0.50	1531	− 0.04 (− 0.24; 0.17)	0.73	1209	0.01 (− 0.21; 0.23)	0.92
Body fat % SDS	698	− 0.02 (− 0.20; 0.16)	0.82	609	− 0.01 (− 0.19; 0.18)	0.94	474	− 0.05 (− 0.26; 0.15)	0.61
WtHR SDS	1610	0.013 (− 0.17; 0.19)	0.89	1452	− 0.077 (− 0.25; 0.10)	0.38	1155	− 0.05 (− 0.24; 0.13)	0.57
Glucose	1602	0.20 (0.06; 0.33)	0.005	1441	0.16 (0.01; 0.31)	0.03	1156	0.20 (0.04; 0.36)	0.016
Insulin	1626	0.08 (− 0.03; 0.19)	0.18	1462	0.05 (− 0.06; 0.17)	0.38	1171	0.09 (− 0.04; 0.22)	0.17
HOMA-IR	1595	0.11 (− 0.01; 0.22)	0.07	1434	0.07 (− 0.05; 0.20)	0.25	1150	0.11 (− 0.03; 0.24)	0.11
HbA1c	1613	0.005 (− 0.0004; 0.01)	0.07	1453	0.003 (− 0.002; 0.01)	0.25	1164	0.06 (− 0.10; 0.22)	0.43
C-peptide	1582	0.06 (− 0.04; 0.17)	0.25	1164	0.06 (− 0.05; 0.17)	0.28	1150	0.10 (− 0.02; 0.22)	0.09

^a^Adjusted for age, sex, BMI SDS (except for the outcome “BMI SDS,” “Body fat % SDS,” and “WtHR SDS”)

^b^Additional adjustment for socioeconomic status

^c^Additional adjustment for puberty stage, defined as pre-pubertal (Tanner stage 1) or pubertal (Tanner stages 2–5)

^*^Sample size after removal of missing values

As a sensitivity analysis, we lowered the cut-off value for *H. pylori* seropositivity to see how it affected the model outputs. For every 10% we lowered the *H. pylori* seropositivity cut-off value, the seroprevalence increased about 2% (Additional file 1: Table S9). The cut-off value could be lowered to 0.7 without changing the logistic and linear regression model outputs markedly (Additional files 1: Table S9 and S10). Excluding subjects with genetic ethnicities other than European resulted in stronger associations between *H. pylori* seropositivity and fasting plasma glucose levels as well as the occurrence of hyperglycemia (Additional files 1: Table S11 and S12). Additional exclusion of subjects with self-reported ethnicity other than Danish led to weaker associations between *H. pylori* seropositivity and both fasting plasma glucose levels and the prevalence of hyperglycemia (Additional files 1: Table S13 and S14).

## Discussion

Opposed to our hypothesis, we did not find an association between *H. pylori* seropositivity and pediatric obesity. Some studies have demonstrated an inverse association between *H. pylori* infection and pediatric obesity [20, 21]; however, our finding aligns with the results of two prior pediatric studies that did not find a correlation: one involving 292 Israeli children with abdominal pain undergoing endoscopy [23] and another including 164 children from South-Korea enrolled for *H. pylori* screening [22]. The underlying reasons for the discrepant results remain uncertain but may arise from factors such as *H. pylori* screening methods, participant selection, inclusion of covariates, or host-microbe interactions influenced by genetic ancestry [15]. Methodological variations are evident across the abovementioned studies related to this topic, with one employing serology [22] and three utilizing histology [20, 21, 23] to determine *H. pylori* infection status. Our findings are based on serology and were not validated by a secondary method such as the urea breath test, as it was conducted after the samples had been collected. However, our study stands out for its notably large sample size of asymptomatic individuals, including a population-based reference cohort, in contrast to the previous studies which focused on smaller cohorts of individuals attending hospital check-ups. The present results also align well with the finding from a large adult cross-sectional study from the United States, where they did not detect any association between *H. pylori* seropositivity and being overweight [57].

A common limitation, but often unavoidable due to pragmatic and economic constraints in many obesity-related studies, is the use of BMI as a proxy for obesity. It has several shortcomings, such as the inability to differentiate between fat, bone, and muscle or the changes in body fat composition depending on age and sex [58]. DEXA offers a solution to the first issue mentioned, but this was mainly performed on the obesity clinic cohort, and only on few individuals from the population-based reference cohort. For the individuals where DEXA was performed, we could not detect an association between *H. pylori* seropositivity and body fat % SDS (Table 3). Waist circumference was available for nearly all study subjects and WtHR has been shown to be a better indicator of abdominal obesity and metabolic disease than BMI in children [59, 60]. However, neither for WtHR SDS did we find an association with *H. pylori* infection status.

Interestingly, *H. pylori* seropositivity was associated with higher prevalence of hyperglycemia and higher concentrations of fasting plasma glucose (Tables 2 and 3). Hyperglycemia is a condition defined as excessive amounts of circulating blood glucose and mainly occurs in people with diabetes. Of note, no individuals diagnosed with diabetes were identified in these subsets of the HOLBAEK study and those with blood glucose and/or HbA1c values in the diabetic range were excluded. The nature of this study does not allow us to speculate about the underlying mechanism driving the observed association between *H. pylori* seropositivity and hyperglycemia, but it has been proposed that *H. pylori* may be contributing to impaired glucose and insulin regulation through modulation of inflammatory and hormonal pathways [61]. *H. pylori* has been shown in a meta-analysis to be a risk factor for type 2 diabetes [62], which most commonly develops in adulthood. However, the increasing obesity rates among children has led to more pediatric type 2 diabetes cases. Additionally, several studies have found a higher prevalence of *H. pylori* among children with type 1 diabetes [63–65]. Another risk factor for diabetes is higher circulating insulin levels, but we did not find any association between *H. pylori* and insulin or pro-insulin (C-peptide) concentrations. Concentrations of HbA1c are commonly used to monitor diabetes treatment but can also be useful in evaluating prediabetes. A large cross-sectional study reported a higher mean level of HbA1c in adults with seropositivity for *H. pylori*, also in a synergistic effect between *H. pylori* and BMI [66]. We found a tendency towards higher HbA1c concentrations in subjects with seropositivity for *H. pylori* after adjusting for sex, age, and BMI SDS*,* but this association disappeared after additional adjustment for socioeconomic status and puberty stage (Table 3).

With our somewhat conservative detection threshold, we found an *H. pylori* prevalence of 14.8% in the study population, with a significantly higher age of individuals with *H. pylori* seropositivity. No recent surveys on *H. pylori* seropositivity in Denmark have been published, but an epidemiologic study conducted from 1982 to 1984 including 4581 adults aged 30–60 years found an overall *H. pylori* prevalence of 25% [67]. In 1998–1999, a population-based *H. pylori* screening disclosed a prevalence of 17% in adults [68]. Hence, our results are in line with an expected overall decrease in prevalence [69]. Several studies have reported low sensitivities when using ELISAs developed for diagnosis of *H. pylori* infection in adults on children [51, 70]. We attempted to adjust our adult-based cut-off value with multiplex serology, but compared to ELISA, the sensitivity was even lower. A possible explanation for this discrepancy could be the use of whole-cell derived antigens in the ELISA, as opposed to the multiplex serology that specifically targets antibodies against 12 selected *H. pylori* antigens. These 12 antigens may be less involved in the early immune response to *H. pylori* in children and adolescents compared to adults.

In human studies, it is difficult to completely control for confounding variables. Our results confirmed that *H. pylori* seropositivity is associated with lower socioeconomic status, as previously reported [71]. This association may be related to ethnicity, as immigrants have a lower socioeconomic status in Denmark [72] and those from non-Western countries would be expected to have a higher *H. pylori* prevalence. Indeed, when we excluded individuals with non-European and non-Danish ethnicities, the association between socioeconomic status and *H. pylori* seropositivity lost significance. We could not detect a significant correlation with sex, although a higher proportion of girls exhibited seropositivity in this study. Previously, a meta-analysis concluded that there is a global male predominance of *H. pylori* infection in adults, but not in children. The authors speculated if this discrepancy is due to differential antibiotic exposure among sexes [73]. A later conducted meta-analysis disclosed a small effect of male sex also in children, but the mechanism by which sex influences *H. pylori* infection remains unknown [74].

Our study exhibits notable strengths and limitations. In contrast to prior reports, the surveyed cohorts cover a large number of individuals with comprehensive sociodemographic and cardiometabolic profiles. Additionally, the study participants were recruited for an obesity study, rather than being selected based on gastroduodenal disease symptoms often related to *H. pylori* infection. One limitation of our study is the absence of data related to birth delivery methods (vaginal or caesarian section) as well as information concerning breast versus formula feeding and antibiotic usage. These factors are recognized contributors to obesity and metabolic disease [75–77]. Additionally, we found a low prevalence of *H. pylori* at 14.8%, which may make it more challenging to detect associations compared to in high prevalence populations. A large proportion of our study population had been genotyped, facilitating the systematic removal of individuals with non-European ethnicity to assess its impact on our analysis. Nonetheless, we acknowledge the possibility that among the subset of subjects with missing genotypes, there may be individuals with non-European ethnicity. Out of the 324 subjects with missing genotypes, 280 had self-reported their ethnicity, and we therefore ran the analyses with additional exclusion of individuals with non-Danish ethnicity. However, this criterion is less objective and may exclude subjects with European ethnicity.

## Conclusions

In conclusion, we find no association between *H. pylori* seropositivity and obesity in Danish children and adolescents. *H. pylori* seropositivity was associated with the incidence of hyperglycemia and higher fasting plasma glucose concentrations. In support of our findings, a recent experimental study revealed that *H. pylori* worsens the impaired glucose regulation in mice on a high-fat diet [78]. Furthermore, our own experimental work on mice fed a high-fat diet showed that early-life *H. pylori* infection worsens the metabolic state and perturbs the distal gut microbiome composition (unpublished results). Taken together, we suggest that the presence of *H. pylori* in children and adolescents can impair glucose metabolism. As such, loss of this ancient microbial associate may be beneficial for early life host metabolism in the Scandinavian population and environment.

## Supplementary Information

Additional file 1: Fig. S1. *Helicobacter pylori* seropositivity (%) in three different age groups. Table S1. Descriptive characteristics of the study population stratified by cohort. Table S2. Multiplex serology *Helicobacter pylori* antigens and antigen-specific cut-offs at 1:100 serum dilution. Table S3. Overview over ELISA and Multiplex serology results. Table S4. Descriptive characteristics of obesity clinic cohort stratified by *Helicobacter pylori* infection status. Table S5. Descriptive characteristics of population-based reference cohort stratified by *Helicobacter pylori* infection status. Table S6. Descriptive characteristics of study population (excluding 71 subjects with non-European ethnicity) stratified by *Helicobacter pylori* infection status. Table S7. Descriptive characteristics of study population (excluding 71 subjects with non-European genetic ethnicity and 159 subjects with self-reported non-Danish ethnicity) stratified by *Helicobacter pylori* infection status. Table S8. Estimated odd ratios (OR) and standardized coefficient (beta) estimates with 95% confidence intervals (CI) for interactions between *Helicobacter pylori* seropositivity and female sex, body mass index standardized deviation score (BMI SDS), socioeconomic status (SES) 2–5 or post- pubertal puberty stage as indicators of hyperglycemia or fasting plasma glucose levels. Table S9. Estimated odd ratios (OR) with 95% confidence intervals (CI) for associations of different cut-off values for *Helicobacter pylori* seropositivity as an indicator of categorical (yes/no) cardiometabolic risk factors. Table S10. Standardized coefficient (beta) estimates with 95% confidence intervals (CI) for associations of different cut-off values for *Helicobacter pylori* seropositivity as an indicator of continuous cardiometabolic risk factors. Table S11. Estimated odd ratios (OR) with 95% confidence intervals (CI) for associations of *Helicobacter pylori* seropositivity as an indicator of categorical (yes/no) cardiometabolic risk factors, excluding 71 subjects with non-European genetic ethnicity. Table S12. Standardized coefficient (beta) estimates with 95% confidence intervals (CI) for associations of *Helicobacter pylori* seropositivity as an indicator of continuous cardiometabolic risk factors, excluding 71 subjects with non-European genetic ethnicity. Table S13. Estimated odd ratios (OR) with 95% confidence intervals (CI) for associations of *Helicobacter pylori* seropositivity as an indicator of categorical (yes/no) cardiometabolic risk factors, excluding 71 subjects with non-European genetic ethnicity and 159 subjects with self-reported non-Danish ethnicity. Table S14. Standardized coefficient (beta) estimates with 95% confidence intervals (CI) for associations of *Helicobacter pylori* seropositivity as an indicator of continuous cardiometabolic risk factors, excluding 71 subjects with non-European genetic ethnicity and 159 subjects with self-reported non-Danish ethnicity.

## Data Availability

Restrictions apply to the availability of the data generated or analyzed during this study to preserve patient confidentiality. The corresponding author will on request detail the restrictions and any conditions under which access to some data may be provided.

## Abbreviations

WHO: World Health Organization
BMI: Body mass index
SDS: Standardized deviation score
HbA1c: Glycated hemoglobin
WtHR: Waist-to-height ratio
DEXA: Dual-energy x-ray absorptiometry
EDTA: Ethylenediaminetetraacetic acid
ELISA: Enzyme-linked immunosorbent assay
PBS: Phosphate-buffered saline
OR: Odds ratio
CI: Confidence interval
