# Supplementary material for: Helicobacter pylori seropositivity associates with hyperglycemia, but not obesity, in Danish children and adolescents
Source: BMC Med. 2024 Sep 11;22:379. doi: 10.1186/s12916-024-03591-w (PMC11389555; doi:10.1186/s12916-024-03591-w)
Supplement: Supplementary file 1 — Additional file 1: Fig. S1. Helicobacter pylori seropositivity (%) in three different age groups. Table S1. Descriptive characteristics of the study population stratified by cohort. Table S2. Multiplex serology Helicobacter pylori antigens and antigen-specific cut-offs at 1:100 serum dilution. Table S3. Overview over ELISA and Multiplex serology results. Table S4. Descriptive characteristics of obesity clinic cohort stratified by Helicobacter pylori infection status. Table S5. Descriptive characteristics of population-based reference cohort stratified by Helicobacter pylori infection status. Table S6. Descriptive characteristics of study population (excluding 71 subjects with non-European ethnicity) stratified by Helicobacter pylori infection status. Table S7. Descriptive characteristics of study population (excluding 71 subjects with non-European genetic ethnicity and 159 subjects with self-reported non-Danish ethnicity) stratified by Helicobacter pylori infection status. Table S8. Estimated odd ratios (OR) and standardized coefficient (beta) estimates with 95% confidence intervals (CI) for interactions between Helicobacter pylori seropositivity and female sex, body mass index standardized deviation score (BMI SDS), socioeconomic status (SES) 2–5 or post- pubertal puberty stage as indicators of hyperglycemia or fasting plasma glucose levels. Table S9. Estimated odd ratios (OR) with 95% confidence intervals (CI) for associations of different cut-off values for Helicobacter pylori seropositivity as an indicator of categorical (yes/no) cardiometabolic risk factors. Table S10. Standardized coefficient (beta) estimates with 95% confidence intervals (CI) for associations of different cut-off values for Helicobacter pylori seropositivity as an indicator of continuous cardiometabolic risk factors. Table S11. Estimated odd ratios (OR) with 95% confidence intervals (CI) for associations of Helicobacter pylori seropositivity as an indicator of categorical (yes/no) cardiometab [file 12916_2024_3591_MOESM1_ESM.pdf]

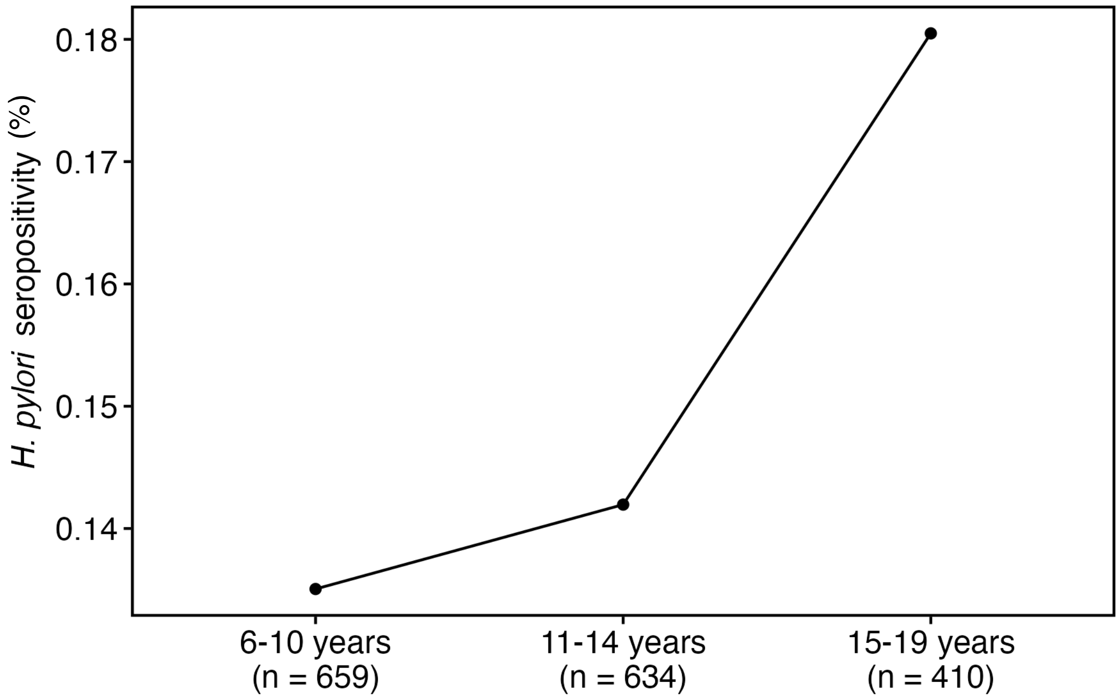

Figure S1: *H. pylori* seropositivity (%) in three different age groups.

Table S1: Descriptive characteristics of the study population stratified by cohort

**Characteristic**		**Cohort[1]** Population-based (n = 990)	Obesity clinic (n = 713)	***p* value[2]**
Age		12.4 (3.7)	12.2 (3.1)	0.5
Sex				<0.001
	male	382 (39%)	346 (49%)	
	female	608 (61%)	367 (51%)	
Socioeconomic status				<0.001
	1	443 (48%)	58 (9.6%)	
	2	220 (24%)	146 (24%)	
	3	189 (20%)	194 (32%)	
	4&5	73 (7.9%)	208 (34%)	
Passive smoking				<0.001
	no	771 (78%)	345 (48%)	
	yes	219 (22%)	368 (52%)	
Puberty stage[3]				<0.001
	pre-pubertal	225 (28%)	222 (41%)	
	post-pubertal	571 (72%)	314 (59%)	
BMI SDS		0.32 (1.06)	2.99 (0.69)	<0.001
Plasma HDL-C (mmol/L)		1.52 (0.33)	1.22 (0.31)	<0.001
Plasma LDL-C (mmol/L)		2.05 (0.62)	2.38 (0.69)	<0.001
Plasma triglycerides (mmol/L)		0.69 (0.32)	1.08 (0.58)	<0.001
Plasma glucose (mmol/L)		5.00 (0.38)	4.95 (0.39)	<0.001
Plasma glucagon (pmol/L)		6.8 (3.5)	9.8 (4.7)	<0.001
Serum insulin (pmol/L)		64 (33)	102 (72)	<0.001
HOMA-IR, (mIU/L)		2.44 (1.34)	3.67 (2.46)	<0.001
Serum C-peptide (nmol/L)		0.59 (0.21)	0.78 (0.40)	<0.001
Whole blood HbA1c (mmol/mol)		33.06 (2.58)	33.37 (2.92)	0.069
Obesity				<0.001
	no	806 (81%)	1 (0.1%)	
	yes	184 (19%)	712 (100%)	
Hyperglycemia				0.061
	no	896 (93%)	570 (90%)	
	yes	68 (7.1%)	62 (9.8%)	
Insulin resistance				<0.001
	no	846 (90%)	424 (68%)	
	yes	90 (9.6%)	198 (32%)	
Dyslipidemia				<0.001
	no	897 (92%)	423 (66%)	
	yes	81 (8.3%)	214 (34%)	
Hypertension				<0.001
	no	949 (96%)	567 (82%)	
	yes	41 (4.1%)	124 (18%)	

[1]Mean (SD); n (%)

[2]Wilcoxon rank sum test; Pearson's Chi-squared test

[3]Puberty stage defined as pre-pubertal (Tanner stage 1) or pubertal (Tanner stage 2-5)

[*]**Abbrevations:** BMI SDS, body mass index standard deviation score; HDL-C, high-density lipoprotein cholesterol; LDL-C, low-density lipoprotein cholesterol; HOMA-IR, homeostasis model assessment of insulin resistance; HbA1c, glycated hemoglobin

Table S2: Multiplex serology *Helicobacter pylori* antigens and antigen-specific cut-offs at 1:100 serum dilution

Name	Systematic name	Acc. No[a]	Selected amino acids	Cut-off [MFI]
H. pylori				
GroEl	HP0010	AM_997163	1-547	400
UreA	HP0073	NP_206873	1-238	1250
HP0231	HP0231	NP_207029	1-265	100
NapA	HP0243	NP_207041	1-144	280
HP0305	HP0305	NP_207103	1-184	150
HpaA	HP0410	NP_207208	1-249	650
CagA N-Terminus	HP0547	NP_207343	1-650	1000
HyuA N-Terminus	HP0695	NP_207208	1-220	600
VacA C-Terminus	HP0887	NP_207680	328-1,008	600
HcpC	HP1098	NP_207889	1-290	200
Cad	HP1104	NP_207895	1-348	270
HP1564	HP1564	NP_208355	1-271	1750

[a]NCBI reference sequence

Table S3: Overview over ELISA and Multiplex serology results. Missing values can be attributed to errors in the bead-counting process.

	ELISA		Multiplex serology
	OD ratio		
***H. pylori* (HP) status[a]**	**mean**	**SD**	**HP proteins[b]**
negative	0.07	0	0
negative	0.04	0	1
negative	0.05	0	0
negative	0.12	0	0
negative	0.18	0	1
negative	0.19	0	1
Intermediate	0.21	0.01	NA
Intermediate	0.23	0	4
Intermediate	0.26	0	4
Intermediate	0.26	0.03	NA
Intermediate	0.3	0.01	4
Intermediate	0.35	0.01	3
Intermediate	0.36	0.02	0
Intermediate	0.36	0.04	3
Intermediate	0.36	0.01	0
Intermediate	0.38	0.01	1
Intermediate	0.41	0.01	3
Intermediate	0.43	0	1
Intermediate	0.45	0.01	2
Intermediate	0.45	0.01	2
Intermediate	0.47	0.06	4
Intermediate	0.47	0.03	1
Intermediate	0.51	0	3
Intermediate	0.52	0.02	1
Intermediate	0.56	0.03	0
Intermediate	0.57	0.02	0
Intermediate	0.6	0.05	0
Intermediate	0.6	0.02	1
Intermediate	0.63	0.03	2
Intermediate	0.67	0.05	0
Intermediate	0.68	0.03	1
Intermediate	0.69	0.01	2
Intermediate	0.7	0	2
Intermediate	0.7	0.01	0
Intermediate	0.72	0.04	0
Intermediate	0.72	0.02	1
Intermediate	0.75	0	0
Intermediate	0.76	0	1
Intermediate	0.76	0.01	2
Intermediate	0.81	0.01	1
Intermediate	0.82	0.02	0
Intermediate	0.84	0.04	3
Intermediate	0.86	0	2
Intermediate	0.93	0.05	NA
Intermediate	0.94	0.06	2
Intermediate	0.94	0.03	NA
Intermediate	0.94	0.01	6
Intermediate	0.96	0.01	1
Intermediate	0.96	0.04	2
Intermediate	0.98	0.01	2
positive	1.18	0.03	NA
positive	1.25	0.05	1
positive	1.34	0.01	0
positive	1.44	0.06	4
positive	1.47	0.03	4
positive	1.56	0.03	0
positive	1.63	0.01	0
positive	1.64	0.05	1
positive	2.16	0.06	0
positive	3.57	0.06	10

[a]positive: OD ratio ≥ 1, intermediate: 1 > OD ratio ≥ 0.2, negative: OD ratio < 0.2;

[b]highlighted in bold font are *H. pylori* multiplex serology positive individuals (positive for >3 proteins)

Table S4: Descriptive characteristics of obesity clinic cohort stratified by *Helicobacter pylori* infection status

		H. pylori[1]		
**Characteristic***		**seronegative (n = 600)**	**seropositive (n = 113)**	***p* value**[2]
Age		12.1 (3.1)	12.5 (3.2)	0.4
Sex				0.3
	male	297 (50%)	49 (43%)	
	female	303 (50%)	64 (57%)	
Socioeconomic status				0.029
	1	46 (9.0%)	12 (13%)	
	2	133 (26%)	13 (14%)	
	3	166 (32%)	28 (30%)	
	4&5	167 (33%)	41 (44%)	
Passive smoking				0.6
	no	287 (48%)	58 (51%)	
	yes	313 (52%)	55 (49%)	
Puberty stage[3]				>0.9
	pre-pubertal	185 (41%)	37 (42%)	
	post-pubertal	262 (59%)	52 (58%)	
BMI SDS		3.00 (0.69)	2.95 (0.71)	0.4
Plasma HDL-C (mmol/L)		1.23 (0.31)	1.20 (0.30)	0.2
Plasma LDL-C (mmol/L)		2.37 (0.70)	2.39 (0.59)	0.4
Plasma triglycerides (mmol/L)		1.08 (0.59)	1.10 (0.49)	0.4
Plasma glucose (mmol/L)		4.94 (0.40)	4.98 (0.36)	0.11
Plasma glucagon (pmol/L)		9.9 (4.6)	9.4 (5.1)	0.2
Serum insulin (pmol/L)		102 (73)	104 (66)	0.2
HOMA-IR, (mIU/L)		3.67 (2.54)	3.67 (1.98)	0.3
Serum C-peptide (nmol/L)		0.77 (0.39)	0.81 (0.44)	0.3
Whole blood HbA1c (mmol/mol)		33.3 (2.9)	33.9 (3.1)	0.2
Obesity				>0.9
	no	1 (0.2%)	0 (0%)	
	yes	599 (100%)	113 (100%)	
Hyperglycemia				0.5
	no	483 (91%)	87 (88%)	
	yes	50 (9.4%)	12 (12%)	
Insulin resistance				0.6
	no	361 (69%)	63 (66%)	
	yes	165 (31%)	33 (34%)	
Dyslipidemia				0.5
	no	354 (66%)	69 (70%)	
	yes	184 (34%)	30 (30%)	
Hypertension				>0.9
	no	477 (82%)	90 (83%)	
	yes	105 (18%)	19 (17%)	

[1]Mean (SD); n (%)

[2]Wilcoxon rank sum test; Pearson's Chi-squared test

[3]Puberty stage defined as pre-pubertal (Tanner stage 1) or pubertal (Tanner stage 2-5)

***Abbrevations:** BMI SDS, body mass index standard deviation score; HDL-C, high-density lipoprotein cholesterol; LDL-C, low-density lipoprotein cholesterol; HOMA-IR, homeostasis model assessment of insulin resistance; HbA1c, glycated hemoglobin

Table S5: Descriptive characteristics of population-based reference cohort stratified by *Helicobacter pylori* infection status

Characteristic*		*H. pylori*[1] seronegative (n = 850)	seropositive (n = 140)	*p* value[2]
Age		12.3 (3.7)	13.0 (3.5)	0.067
Sex				0.4
	male	333 (39%)	49 (35%)	
	female	517 (61%)	91 (65%)	
Socioeconomic status				0.3
	1	384 (48%)	59 (46%)	
	2	193 (24%)	27 (21%)	
	3	161 (20%)	28 (22%)	
	4&5	58 (7.3%)	15 (12%)	
Passive smoking				0.021
	no	673 (79%)	98 (70%)	
	yes	177 (21%)	42 (30%)	
Puberty stage[3]				0.074
	pre-pubertal	202 (29%)	23 (21%)	
	post-pubertal	483 (71%)	88 (79%)	
BMI SDS		0.33 (1.08)	0.28 (0.94)	0.7
Plasma HDL-C (mmol/L)		1.52 (0.33)	1.53 (0.35)	0.8
Plasma LDL-C (mmol/L)		2.04 (0.62)	2.08 (0.64)	0.5
Plasma triglycerides (mmol/L)		0.69 (0.32)	0.68 (0.33)	0.8
Plasma glucose (mmol/L)		4.99 (0.38)	5.09 (0.37)	0.014
Plasma glucagon (pmol/L)		6.8 (3.6)	6.7 (3.2)	0.9
Serum insulin (pmol/L)		64 (33)	67 (30)	0.076
HOMA-IR, (mIU/L)		2.41 (1.35)	2.61 (1.26)	0.034
Serum C-peptide (nmol/L)		0.58 (0.21)	0.61 (0.20)	0.2
Whole blood HbA1c (mmol/mol)		33.04 (2.56)	33.17 (2.68)	0.8
Obesity				0.13
	no	685 (81%)	121 (86%)	
	yes	165 (19%)	19 (14%)	
Hyperglycemia				<0.001
	no	783 (94%)	113 (85%)	
	yes	48 (5.8%)	20 (15%)	
Insulin resistance				0.5
	no	731 (91%)	115 (88%)	
	yes	75 (9.3%)	15 (12%)	
Dyslipidemia				>0.9
	no	772 (92%)	125 (92%)	
	yes	70 (8.3%)	11 (8.1%)	
Hypertension				0.7
	no	816 (96%)	133 (95%)	
	yes	34 (4.0%)	7 (5.0%)	

[1]Mean (SD); n (%)

[2]Wilcoxon rank sum test; Pearson's Chi-squared test

[3]Puberty stage defined as pre-pubertal (Tanner stage 1) or pubertal (Tanner stage 2-5)

***Abbrevations:** BMI SDS, body mass index standard deviation score; HDL-C, high-density lipoprotein cholesterol; LDL-C, low-density lipoprotein cholesterol; HOMA-IR, homeostasis model assessment of insulin resistance; HbA1c, glycated hemoglobin

Table S6: Descriptive characteristics of study population stratified by *Helicobacter pylori* infection status. 71 subjects with non-European ethnicity were excluded.

**Characteristic***		***H. pylori*[1]** seronegative (n = 1398)	**seropositive (n = 234)**	***p* value[2]**
Age		12.3 (3.5)	12.7 (3.4)	0.075
Sex				0.14
	male	601 (43%)	88 (38%)	
	female	797 (57%)	146 (62%)	
Socioeconomic status				0.083
	1	425 (33%)	70 (33%)	
	2	314 (25%)	39 (18%)	
	3	325 (26%)	56 (26%)	
	4&5	208 (16%)	47 (22%)	
Passive smoking				0.2
	no	928 (66%)	144 (62%)	
	yes	470 (34%)	90 (38%)	
Puberty stage[3]				0.2
	pre-pubertal	374 (34%)	56 (30%)	
	post-pubertal	716 (66%)	133 (70%)	
BMI SDS		1.40 (1.61)	1.35 (1.55)	0.6
Plasma HDL-C (mmol/L)		1.40 (0.35)	1.40 (0.37)	0.5
Plasma LDL-C (mmol/L)		2.17 (0.67)	2.21 (0.64)	0.3
Plasma triglycerides (mmol/L)		0.84 (0.49)	0.84 (0.45)	0.8
Plasma glucose (mmol/L)		4.97 (0.39)	5.05 (0.37)	0.003
Plasma glucagon (pmol/L)		7.9 (4.2)	7.7 (3.9)	0.4
Serum insulin (pmol/L)		78 (54)	81 (52)	0.055
HOMA-IR, (mIU/L)		2.87 (2.00)	2.99 (1.67)	0.022
Serum C-peptide (nmol/L)		0.65 (0.30)	0.68 (0.34)	0.2
Whole blood HbA1c (mmol/mol)		33.09 (2.66)	33.38 (2.86)	0.3
Obesity				0.5
	no	675 (48%)	119 (51%)	
	yes	723 (52%)	115 (49%)	
Hyperglycemia				<0.001
	no	1,224 (93%)	184 (86%)	
	yes	91 (6.9%)	30 (14%)	
Insulin resistance				0.4
	no	1,062 (83%)	167 (80%)	
	yes	221 (17%)	42 (20%)	
Dyslipidemia				0.5
	no	1,086 (82%)	182 (84%)	
	yes	244 (18%)	35 (16%)	
Hypertension				0.8
	no	1,247 (90%)	210 (91%)	
	yes	136 (9.8%)	21 (9.1%)	

[1]Mean (SD); n (%)

[2]Wilcoxon rank sum test; Pearson's Chi-squared test

[3]Puberty stage defined as pre-pubertal (Tanner stage 1) or pubertal (Tanner stage 2-5)

***Abbrevations:** BMI SDS, body mass index standard deviation score; HDL-C, high-density lipoprotein cholesterol; LDL-C, low-density lipoprotein cholesterol; HOMA-IR, homeostasis model assessment of insulin resistance; HbA1c, glycated hemoglobin

Table S7: Descriptive characteristics of study population stratified by *Helicobacter pylori* infection status. 71 subjects with non-European genetic ethnicity and 159 subjects with self-reported non-Danish ethnicity were excluded.

Characteristic*		*H. pylori*[1]		
		**seronegative (n = 1276)**	**seropositive (n = 197)**	***p* value**[2]
Age		12.3 (3.5)	12.6 (3.4)	0.4
Sex				0.077
	male	549 (43%)	71 (36%)	
	female	727 (57%)	126 (64%)	
Socioeconomic status				0.11
	1	404 (35%)	63 (34%)	
	2	296 (25%)	35 (19%)	
	3	301 (26%)	49 (27%)	
	4&5	169 (14%)	37 (20%)	
Passive smoking				0.12
	no	866 (68%)	122 (62%)	
	yes	410 (32%)	75 (38%)	
Puberty stage[3]				0.4
	pre-pubertal	341 (34%)	50 (31%)	
	post-pubertal	659 (66%)	113 (69%)	
BMI SDS		1.35 (1.61)	1.23 (1.51)	0.3
Plasma HDL-C (mmol/L)		1.40 (0.35)	1.42 (0.37)	>0.9
Plasma LDL-C (mmol/L)		2.16 (0.67)	2.17 (0.65)	0.9
Plasma triglycerides (mmol/L)		0.83 (0.48)	0.81 (0.44)	0.7
Plasma glucose (mmol/L)		4.96 (0.39)	5.03 (0.36)	0.037
Plasma glucagon (pmol/L)		7.9 (4.3)	7.5 (3.8)	0.2
Serum insulin (pmol/L)		75 (51)	73 (38)	0.6
HOMA-IR, (mIU/L)		2.81 (1.98)	2.77 (1.50)	0.3
Serum C-peptide (nmol/L)		0.64 (0.28)	0.64 (0.24)	0.5
Whole blood HbA1c (mmol/mol)		33.08 (2.62)	33.05 (2.66)	0.7
Obesity				0.3
	no	633 (50%)	106 (54%)	
	yes	643 (50%)	91 (46%)	
Hyperglycemia				0.12
	no	1,123 (93%)	163 (90%)	
	yes	83 (6.9%)	19 (10%)	
Insulin resistance				>0.9
	no	985 (84%)	148 (84%)	
	yes	190 (16%)	29 (16%)	
Dyslipidemia				0.5
	no	999 (82%)	156 (84%)	
	yes	220 (18%)	29 (16%)	
Hypertension				0.5
	no	1,139 (90%)	179 (92%)	
	yes	122 (9.7%)	15 (7.7%)	

[1]Mean (SD); n (%)

[2]Wilcoxon rank sum test; Pearson's Chi-squared test

[3]Puberty stage defined as pre-pubertal (Tanner stage 1) or pubertal (Tanner stage 2-5)

***Abbrevations:** BMI SDS, body mass index standard deviation score; HDL-C, high-density lipoprotein cholesterol; LDL-C, low-density lipoprotein cholesterol; HOMA-IR, homeostasis model assessment of insulin resistance; HbA1c, glycated hemoglobin

Table S8: Estimated odd ratios (OR) and standardized coefficient (beta) estimates with 95 % confidence intervals (CI) for interations between *Helicobacter pylori* seropositivity and female sex, body mass index standardized devation score (BMI SDS), socioeconomic status (SES) 2-5 or post-pubertal puberty stage as indicators of hyperglycemia or fasting plasma glucose levels. Fasting plasma glucose levels were log10-transformed and z-scored.

**Characteristic**	**model***	**Interaction**	**OR**	**95% CI**	***p* value**
Hyperglycemia	1	HP_pos * BMI_SDS	0.88	0.67, 1.16	0.4
Hyperglycemia	2	HP_pos * BMI_SDS	0.88	0.66, 1.18	0.4
Hyperglycemia	2	HP_pos * SES2	0.74	0.16, 3.07	0.7
Hyperglycemia	2	HP_pos * SES3	1.41	0.38, 5.32	0.6
Hyperglycemia	2	HP_pos * SES4&5	0.66	0.19, 2.32	0.5
Hyperglycemia	3	HP_pos * post_pubertal	1.1	0.35, 3.98	0.9
Hyperglycemia	3	HP_pos * sex_female	0.77	0.27, 2.21	0.6
Hyperglycemia	3	HP_pos * BMI_SDS	0.86	0.63, 1.19	0.4
Hyperglycemia	3	HP_pos * SES2	0.75	0.16, 3.26	0.7
Hyperglycemia	3	HP_pos * SES3	1.21	0.29, 5.04	0.8
Hyperglycemia	3	HP_pos * SES4&5	0.69	0.18, 2.63	0.6
**Characteristic**	**model***	**Interaction**	**beta**	**95% CI**	***p* value**
Glucose	1	HP_pos * sex_female	0.97	0.73, 1.28	0.8
Glucose	2	HP_pos * SES2	-0.13	-0.55, 0.29	0.5
Glucose	2	HP_pos * SES3	-0.12	-0.51, 0.27	0.5
Glucose	2	HP_pos * SES4&5	-0.07	-0.47, 0.32	0.7
Glucose	2	HP_pos * sex_female	-0.05	-0.35, 0.25	0.7
Glucose	3	HP_pos * sex_female	-0.12	-0.47, 0.23	0.5
Glucose	3	HP_pos * post_pubertal	0.01	-0.34, 0.36	>0.9
Glucose	3	HP_pos * SES2	-0.27	-0.73, 0.20	0.3
Glucose	3	HP_pos * SES3	-0.22	-0.65, 0.20	0.3
Glucose	3	HP_pos * SES4&5	-0.07	-0.50, 0.37	0.8

*model 1 adjusted for age, sex, BMI SDS

model 2: model 1 + additional adjustment for socioeconomic status

model 3: model 1 + model 2 + additional adjustment for puberty stage

Table S9: Estimated odd ratios (OR) with 95 % confidence intervals (CI) for associations of different cut-off values for *Helicobacter pylori* seropositivity as an indicator of categorical (yes/no) cardiometabolic risk factors.

cutoff	outcome	n	OR	95 % CI		*p* value	model*	*H. pylori* seropositivity
				lower	upper			
0.9	Obesity	1703	1.061	0.820	1.374	0.655	1	16.4
0.9	Obesity	1531	0.925	0.682	1.254	0.616	2	16.4
0.9	Obesity	1209	1.023	0.724	1.446	0.895	3	16.4
0.9	Hyperglycemia	1596	1.880	1.219	2.838	0.003	1	16.4
0.9	Hyperglycemia	1436	1.819	1.135	2.844	0.010	2	16.4
0.9	Hyperglycemia	1151	2.231	1.346	3.615	0.001	3	16.4
0.9	Hypertension	1681	1.020	0.644	1.566	0.931	1	16.4
0.9	Hypertension	1514	0.830	0.491	1.345	0.467	2	16.4
0.9	Hypertension	1199	0.951	0.547	1.583	0.851	3	16.4
0.9	Dyslipidemia	1615	0.855	0.582	1.237	0.415	1	16.4
0.9	Dyslipidemia	1455	0.879	0.579	1.310	0.536	2	16.4
0.9	Dyslipidemia	1164	0.785	0.488	1.231	0.303	3	16.4
0.9	Insulin resistance	1558	1.302	0.898	1.869	0.158	1	16.4
0.9	Insulin resistance	1404	1.189	0.789	1.769	0.400	2	16.4
0.9	Insulin resistance	1122	1.430	0.908	2.224	0.116	3	16.4
0.8	Obesity	1703	1.023	0.798	1.313	0.856	1	18.1
0.8	Obesity	1531	0.909	0.678	1.218	0.522	2	18.1
0.8	Obesity	1209	0.920	0.658	1.284	0.623	3	18.1
0.8	Hyperglycemia	1596	1.749	1.141	2.627	0.008	1	18.1
0.8	Hyperglycemia	1436	1.692	1.063	2.631	0.022	2	18.1
0.8	Hyperglycemia	1151	2.002	1.211	3.233	0.005	3	18.1
0.8	Hypertension	1681	0.999	0.640	1.517	0.995	1	18.1
0.8	Hypertension	1514	0.826	0.499	1.317	0.440	2	18.1
0.8	Hypertension	1199	0.993	0.584	1.626	0.977	3	18.1
0.8	Dyslipidemia	1615	0.919	0.637	1.310	0.647	1	18.1
0.8	Dyslipidemia	1455	0.930	0.624	1.364	0.714	2	18.1
0.8	Dyslipidemia	1164	0.802	0.505	1.243	0.335	3	18.1
0.8	Insulin resistance	1558	1.284	0.895	1.825	0.169	1	18.1
0.8	Insulin resistance	1404	1.175	0.789	1.729	0.418	2	18.1
0.8	Insulin resistance	1122	1.348	0.861	2.083	0.184	3	18.1
0.7	Obesity	1703	0.994	0.785	1.260	0.962	1	20.6
0.7	Obesity	1531	0.905	0.685	1.194	0.481	2	20.6
0.7	Obesity	1209	0.890	0.647	1.222	0.471	3	20.6
0.7	Hyperglycemia	1596	1.623	1.074	2.411	0.019	1	20.6
0.7	Hyperglycemia	1436	1.599	1.022	2.454	0.035	2	20.6
0.7	Hyperglycemia	1151	1.960	1.208	3.123	0.005	3	20.6
0.7	Hypertension	1681	1.058	0.699	1.568	0.784	1	20.6
0.7	Hypertension	1514	0.941	0.594	1.448	0.787	2	20.6
0.7	Hypertension	1199	1.155	0.711	1.827	0.549	3	20.6
0.7	Dyslipidemia	1615	1.053	0.749	1.464	0.764	1	20.6
0.7	Dyslipidemia	1455	1.118	0.776	1.594	0.542	2	20.6
0.7	Dyslipidemia	1164	1.010	0.665	1.512	0.962	3	20.6
0.7	Insulin resistance	1558	1.157	0.819	1.622	0.401	1	20.6
0.7	Insulin resistance	1404	1.048	0.716	1.519	0.805	2	20.6
0.7	Insulin resistance	1122	1.252	0.815	1.899	0.297	3	20.6

*model 1 adjusted for age, sex, BMI SDS (except for the outcome "Obesity")
model 2: model 1 + additional adjustment for socioeconomic status
model 3: model 1 + model 2 + additional adjustment for puberty stage

Table S10: Standardized coefficient (beta) estimates with 95 % confidence intervals (CI) for associations of different cut-off values for *Helicobacter pylori* seropositivity as an indicator of continuous cardiometabolic risk factors. Outcome variables were log10-transformed and z-scored, except for BMI SDS, bodyfat % SDS and WtHR SDS.

				95 % CI			
**cutoff**	**outcome**	**n**	**beta**	**lower**	**upper**	***p* value**	**model**
0.9	BMI SDS	1703	0.092	-0.111	0.295	0.373	1
0.9	BMI SDS	1531	0.000	-0.192	0.193	0.997	2
0.9	BMI SDS	1209	0.050	-0.160	0.259	0.641	3
0.9	Body fat % SDS	698	-0.021	-0.204	0.161	0.819	1
0.9	Body fat % SDS	609	0.010	-0.165	0.185	0.910	2
0.9	Body fat % SDS	474	-0.039	-0.234	0.156	0.693	3
0.9	WtHR SDS	1610	0.050	-0.122	0.223	0.567	1
0.9	WtHR SDS	1452	-0.032	-0.196	0.132	0.703	2
0.9	WtHR SDS	1155	-0.013	-0.194	0.169	0.892	3
0.9	Glucose	1602	0.162	0.031	0.293	0.015	1
0.9	Glucose	1441	0.128	-0.011	0.267	0.070	2
0.9	Glucose	1156	0.191	0.038	0.344	0.015	3
0.9	Insulin	1626	0.069	-0.037	0.176	0.203	1
0.9	Insulin	1462	0.042	-0.069	0.153	0.456	2
0.9	Insulin	1171	0.078	-0.044	0.200	0.210	3
0.9	HOMA	1595	0.097	-0.014	0.209	0.088	1
0.9	HOMA	1434	0.059	-0.058	0.176	0.326	2
0.9	HOMA	1150	0.100	-0.028	0.228	0.126	3
0.9	HbA1c	1613	0.004	0.000	0.009	0.076	1
0.9	HbA1c	1453	0.003	-0.002	0.008	0.256	2
0.9	HbA1c	1164	0.065	-0.085	0.216	0.396	3
0.9	C-peptide	1582	0.065	-0.037	0.167	0.214	1
0.9	C-peptide	1423	0.059	-0.047	0.165	0.278	2
0.9	C-peptide	1150	0.097	-0.017	0.211	0.096	3
0.8	BMI SDS	1703	0.055	-0.141	0.251	0.580	1
0.8	BMI SDS	1531	-0.028	-0.213	0.157	0.767	2
0.8	BMI SDS	1209	-0.025	-0.227	0.178	0.810	3
0.8	Body fat % SDS	698	-0.021	-0.204	0.161	0.819	1
0.8	Body fat % SDS	609	0.017	-0.153	0.188	0.842	2
0.8	Body fat % SDS	474	-0.032	-0.223	0.160	0.744	3
0.8	WtHR SDS	1610	0.018	-0.148	0.185	0.828	1
0.8	WtHR SDS	1452	-0.054	-0.212	0.104	0.502	2
0.8	WtHR SDS	1155	-0.068	-0.243	0.107	0.447	3
0.8	Glucose	1602	0.181	0.055	0.307	0.005	1
0.8	Glucose	1441	0.156	0.022	0.290	0.022	2
0.8	Glucose	1156	0.208	0.060	0.356	0.006	3
0.8	Insulin	1626	0.070	-0.033	0.174	0.181	1
0.8	Insulin	1462	0.052	-0.055	0.159	0.339	2
0.8	Insulin	1171	0.074	-0.044	0.192	0.222	3
0.8	HOMA	1595	0.101	-0.007	0.208	0.066	1
0.8	HOMA	1434	0.073	-0.040	0.185	0.207	2
0.8	HOMA	1150	0.098	-0.026	0.222	0.122	3
0.8	HbA1c	1613	0.004	0.000	0.009	0.077	1
0.8	HbA1c	1453	0.003	-0.002	0.008	0.209	2
0.8	HbA1c	1164	0.072	-0.074	0.217	0.333	3
0.8	C-peptide	1582	0.074	-0.024	0.173	0.140	1
0.8	C-peptide	1423	0.075	-0.028	0.177	0.154	2
0.8	C-peptide	1150	0.101	-0.009	0.212	0.072	3
0.7	BMI SDS	1703	0.054	-0.132	0.241	0.566	1
0.7	BMI SDS	1531	-0.002	-0.177	0.174	0.986	2
0.7	BMI SDS	1209	-0.013	-0.206	0.179	0.892	3
0.7	Body fat % SDS	698	-0.021	-0.204	0.161	0.819	1
0.7	Body fat % SDS	609	0.014	-0.148	0.175	0.870	2
0.7	Body fat % SDS	474	-0.041	-0.222	0.140	0.658	3
0.7	WtHR SDS	1610	0.029	-0.129	0.187	0.720	1
0.7	WtHR SDS	1452	-0.030	-0.180	0.121	0.700	2
0.7	WtHR SDS	1155	-0.054	-0.220	0.113	0.528	3
0.7	Glucose	1602	0.129	0.010	0.249	0.034	1
0.7	Glucose	1441	0.105	-0.022	0.231	0.104	2
0.7	Glucose	1156	0.171	0.031	0.311	0.017	3
0.7	Insulin	1626	0.047	-0.051	0.145	0.345	1
0.7	Insulin	1462	0.028	-0.074	0.129	0.590	2
0.7	Insulin	1171	0.063	-0.049	0.175	0.270	3
0.7	HOMA	1595	0.072	-0.030	0.173	0.169	1
0.7	HOMA	1434	0.042	-0.064	0.149	0.438	2
0.7	HOMA	1150	0.084	-0.033	0.201	0.161	3
0.7	HbA1c	1613	0.001	-0.003	0.005	0.637	1
0.7	HbA1c	1453	0.001	-0.004	0.005	0.782	2
0.7	HbA1c	1164	0.003	-0.135	0.141	0.966	3
0.7	C-peptide	1582	0.053	-0.040	0.146	0.266	1
0.7	C-peptide	1423	0.050	-0.047	0.147	0.308	2
0.7	C-peptide	1150	0.084	-0.021	0.189	0.117	3

*model 1 adjusted for age, sex, BMI SDS (except for the outcome "BMI SDS", "Body fat % SDS" and "WtHR SDS")
model 2: model 1 + additional adjustment for socioeconomic status
model 3: model 1 + model 2 + additional adjustment for puberty stage

Table S11: Estimated odd ratios (OR) with 95 % confidence intervals (CI) for associations of *Helicobacter pylori* seropositivity as an indicator of categorical (yes/no) cardiometabolic risk factors. 71 subjects with non-European genetic ethnicity were excluded.

	Model 1[a]			Model 2[b]			Model 3[c]		
	**n***	**OR (95% CI)**	**p value**	**n***	**OR (95% CI)**	**p value**	**n***	**OR (95% CI)**	**p value**
Obesity	1632	0.93 (0.70;1.23)	0.61	1484	0.83 (0.60;1.15)	0.28	1170	0.94 (0.65;1.36)	0.75
Hyperglycemia	1529	2.21 (1.40;3.42)	0.0005	1391	2.11 (1.28;3.37)	0.002	1114	2.49 (1.46;4.14)	0.001
Hypertension	1614	0.97 (0.57;1.56)	0.89	1468	0.79 (0.44;1.35)	0.41	1161	0.84 (0.45;1.47)	0.55
Dyslipidemia	1547	0.86 (0.56;1.29)	0.47	1409	0.83 (0.52;1.29)	0.42	1126	0.70 (0.41;1.15)	0.17
Insulin resistance	1492	1.34 (0.89;2.00)	0.16	1359	1.25 (0.80;1.93)	0.31	1085	1.49 (0.92;2.39)	0.10

[a]adjusted for age, sex, BMI SDS (except for the outcome "obesity")
[b]additional adjustment for socioeconomic status
[c]additional adjustment for puberty stage
*sample size after removal of missing values

Table S12: Standardized coefficient (beta) estimates with 95 % confidence intervals (CI) for associations of *Helicobacter pylori* seropositivity as an indicator of continuous cardiometabolic risk factors. Outcome variables were log10-transformed and z-scored except for body mass index (BMI) standard deviation score (SDS), bodyfat % SDS and waist to height ratio (WtHR) SDS. 71 subjects with non-European genetic ethnicity were excluded.

	Model 1[a]			Model 2[b]			Model 3[c]		
	**n***	**beta (95% CI)**	***p***	**n***	**beta (95% CI)**	***p***	**n***	**beta (95% CI)**	***p***
BMI SDS	1632	-0.02 (-0.24;0.20)	0.88	1484	-0.06 (-0.27;0.14)	0.56	1170	0.01 (-0.21;0.24)	0.92
Body fat (%) SDS	649	-0.05 (-0.24;0.14)	0.61	573	0.00 (-0.19;0.20)	0.98	442	-0.03 (-0.24;0.18)	0.79
WtHR SDS	1546	-0.063 (-0.25;0.12)	0.51	1408	-0.105 (-0.28;0.07)	0.25	1118	-0.06 (-0.25;0.13)	0.55
Glucose	1535	0.22 (0.08;0.37)	0.002	1396	0.18 (0.03;0.33)	0.017	1119	0.22 (0.06;0.38)	0.009
Insulin	1557	0.08 (-0.03;0.20)	0.16	1416	0.06 (-0.06;0.18)	0.34	1133	0.09 (-0.04;0.22)	0.18
HOMA-IR	1528	0.12 (-0.01;0.24)	0.06	1389	0.08 (-0.05;0.21)	0.22	1113	0.11 (-0.03;0.25)	0.11
HbA1c	1545	0.004 (-0.001;0.01)	0.10	1407	0.003 (-0.003;0.008)	0.32	1126	0.07 (-0.10;0.23)	0.42
C-peptide	1515	0.064 (-0.0474;0.17)	0.26	1379	0.06 (-0.06;0.18)	0.31	1114	0.11 (-0.02;0.23)	0.09

[a]adjusted for age, sex, BMI SDS (except for the outcome "BMI SDS", "Bodyfat % SDS" and "WtHR SDS")
[b]additional adjustment for socioeconomic status
[c]additional adjustment for puberty stage
*sample size after removal of missing values

Table S13: Estimated odd ratios (OR) with 95 % confidence intervals (CI) for associations of *Helicobacter pylori* seropositivity as an indicator of categorical (yes/no) cardiometabolic risk factors. 71 subjects with non-European genetic ethnicity and 159 subjects with self-reported non-Danish ethnicity were excluded.

	Model 1[a]			Model 2[b]			Model 3[c]		
	**n***	**OR (95% CI)**	**p value**	**n***	**OR (95% CI)**	**p value**	**n***	**OR (95% CI)**	**p value**
Obesity	1473	0.87 (0.64;1.18)	0.36	1354	0.75 (0.53;1.06)	0.10	1073	0.83 (0.56;1.22)	0.35
Hyperglycemia	1388	1.64 (0.94;2.74)	0.07	1275	1.58 (0.87;2.72)	0.12	1025	2.01 (1.09;3.55)	0.02
Hypertension	1455	0.85 (0.46;1.47)	0.58	1338	0.80 (0.41;1.43)	0.47	1064	0.81 (0.40;1.50)	0.53
Dyslipidemia	1404	0.92 (0.57;1.43)	0.71	1291	0.93 (0.56;1.49)	0.76	1036	0.78 (0.44;1.32)	0.36
Insulin resistance	1352	1.15 (0.72;1.81)	0.54	1244	1.10 (0.67;1.77)	0.69	997	1.26 (0.73;2.11)	0.39

[a]adjusted for age, sex, BMI SDS (except for the outcome "obesity")
[b]additional adjustment for socioeconomic status
[c]additional adjustment for puberty stage
[*]sample size after removal of missing values

Table S14: Standardized coefficient (beta) estimates with 95 % confidence intervals (CI) for associations of *Helicobacter pylori* seropositivity as an indicator of continuous cardiometabolic risk factors. Outcome variables were log10-transformed and z-scored except for body mass index (BMI) standard deviation score (SDS), bodyfat % SDS and waist to height ratio (WtHR) SDS. 71 subjects with non-European genetic ethnicity and 159 subjects with self-reported non-Danish ethnicity were excluded.

	Model 1[a]			Model 2[b]			Model 3[c]		
	**n***	**beta (95% CI)**	***p***	**n***	**beta (95% CI)**	***p***	**n***	**beta (95% CI)**	***p***
BMI SDS	1473	-0.08 (-0.32;0.16)	0.51	1354	-0.15 (-0.37;0.07)	0.19	1073	-0.07 (-0.31;0.17)	0.56
Body fat (%) SDS	580	-0.08 (-0.30;0.13)	0.44	514	-0.02 (-0.23;0.19)	0.86	398	-0.04 (-0.27;0.19)	0.71
WtHR SDS	1396	-0.095 (-0.30;0.11)	0.35	1286	-0.153 (-0.34;0.04)	0.11	1024	-0.10 (-0.31;0.10)	0.32
Glucose	1393	0.19 (0.03;0.34)	0.017	1279	0.17 (0.01;0.33)	0.04	1029	0.22 (0.05;0.39)	0.013
Insulin	1410	0.02 (-0.11;0.15)	0.76	1296	0.01 (-0.13;0.14)	0.93	1041	0.03 (-0.11;0.17)	0.70
HOMA-IR	1387	0.06 (-0.07;0.19)	0.35	1273	0.05 (-0.09;0.18)	0.50	1024	0.08 (-0.07;0.22)	0.31
HbA1c	1403	0.0001 (-0.005;0.01)	0.98	1290	-0.001 (-0.006;0.005)	0.79	1037	-0.02 (-0.19;0.16)	0.85
C-peptide	1373	0.027 (-0.0932;0.15)	0.66	1264	0.02 (-0.11;0.14)	0.79	1025	0.05 (-0.08;0.18)	0.42

[a]adjusted for age, sex, BMI SDS (except for the outcome "BMI SDS", "Bodyfat % SDS" and "WtHR SDS")
[b]additional adjustment for socioeconomic status
[c]additional adjustment for puberty stage
[*]sample size after removal of missing values
